# Immunocompromised Child on Infliximab: A Case Report of *Listeria monocytogenes* Meningitis

**DOI:** 10.5811/cpcem.2020.7.48053

**Published:** 2020-09-09

**Authors:** Camille Halfman, Luke Slate, Julienne Yamamoto, Samantha Jones

**Affiliations:** SUNY Upstate Medical University, Department of Emergency Medicine, Syracuse, New York

**Keywords:** listeria monocytogenes, infliximab

## Abstract

**Introduction:**

Patients with naturally occurring, impaired cell-mediated immunity secondary to age and pregnancy are known to be at risk of developing severe and invasive *Listeria monocytogenes* infections. Immunosuppressant medications, particularly infliximab, are also known to increase this risk.

**Case Report:**

We present the case of a seven-year-old female on infliximab who was diagnosed with culture positive *L. monocytogenes* meningitis after a negative cerebral spinal fluid polymerase chain reaction (PCR).

**Conclusion:**

Patients receiving infliximab who display signs of central nervous system infection should be suspected to have *L. monocytogenes* as an infecting agent, and empiric addition of ampicillin to their antibiotic regimen should be considered, with substitution of trimethoprim-sulfamethoxazole in cases of penicillin allergy, regardless of initial PCR results.

## INTRODUCTION

*Listeria monocytogene*s is a facultative anaerobic, gram-positive bacillus found in the intestinal flora of many animals and humans, and is spread through contaminated foods such as unpasteurized milk, soft cheeses, undercooked meats, and raw vegetables. It is almost universally susceptible to ampicillin but, if left untreated, it can cause serious infection with a propensity for sepsis, pneumonia, and meningoencephalitis. It has also been documented to cause acute hydrocephalus secondary to invasion of the brainstem and is often fatal.^13^

Patients with naturally occurring, impaired cell-mediated immunity secondary to extremes of age and pregnancy are known to be at risk of developing severe and invasive *L. monocytogenes* infections. These populations are routinely advised to avoid potential sources of *L. monocytogenes* and are empirically treated with ampicillin when severe bacterial infection is suspected. Due to the significant mortality of listeriosis infection and the fact that it is not well covered by standard empiric antibiotic treatment, a high index of suspicion for this organism should be maintained.^3^

A number of listeriosis cases in adults who are immunocompromised secondary to infliximab use have been reported.^1^,^11^ Infliximab is a tumor necrosis factor-α (TNF-α) inhibitor commonly used in treatment of rheumatoid arthritis and Crohn’s disease. TNF-α is a protein produced by monocytes, macrophages, B-cells, T-cells, and fibroblasts to stimulate proliferation of cytokines and inflammatory cascades. Immunosuppression secondary to TNF-α inhibitor therapy is a known risk factor for Listeria infection, 4, 5 although the American Academy of Pediatrics has not yet recommended empiric coverage in patients receiving TNF-α inhibitor therapy. Here, we present a seven-year-old female on infliximab therapy who developed listeria meningitis despite a negative polymerase chain reaction (PCR) assay of cerebral spinal fluid (CSF).

## CASE REPORT

A seven-year-old female presented to the emergency department (ED) for fever and headache for one day. She was reportedly very tired after returning from school and took a nap. Upon waking, she complained of a headache and had a documented fever of 40° Celsius. Shortly after taking ibuprofen, she had an episode of emesis and subsequently sought medical attention. In the ED, she reported headache, decreased appetite, photophobia, and nausea. She had received induction of infliximab 20 days prior to presentation after a diagnosis of Crohn’s disease, and had her second infusion two days prior. She was also taking prednisone daily. She was tachycardic, febrile, uncomfortable-appearing and photophobic. She had no focal neurologic deficits. She did display nuchal rigidity, but Kernig’s and Brudzinski’s signs were negative.

Blood work was obtained and the patient was given 20 milliliters per kilogram normal saline bolus with minimal improvement in tachycardia. The patient’s white blood cell count was 13.7 × 10^3^/ microliter (μL) (reference range 4.5–13 × 10^3^/μL), absolute neutrophil count 8.79 × 10^3^/μL (reference range 1.5 – 8.0 × 10^3^/μL), and urinalysis was within normal limits. Blood and urine cultures were obtained, and a respiratory viral panel returned positive for coronavirus.

Empiric treatment of suspected meningitis was initiated with vancomycin and ceftriaxone. Lumbar puncture was performed under ketamine sedation and the patient was admitted to the pediatric hospitalist service. CSF analysis showed 2025 total nucleated cells (90% neutrophils, reference range < 7) and 6 red blood cells (RBCs)/μL (reference range < 2) in Tube 1; 1640 total nucleated cells (92% neutrophils) and 6 RBCs/ μL in Tube 3. CSF glucose was 56 milligrams per deciliter (mg/dL) (reference range 60 – 80 mg/dL), protein was 99 mg/dL (reference range 15 – 45 mg/dL). Gram stain showed +4 white blood cells but no organisms noted. Shortly after admission, CSF PCR returned negative for *L. monocytogenes*, *E. coli K1*, *H. influenzae*, *N. meningitidis*, *S. agalactiae*, *S. pneumoniae*, *C. neoformans/gatti*, cytomegalovirus, enterovirus, herpes simplex virus 1 and 2, human herpesvirus 6, human parechovirus, and varicella zoster virus.

Upon admission, the patient’s immunosuppressive medications were discontinued. Antibiotic coverage was not broadened from vancomycin and ceftriaxone owing to the negative CSF PCR. She continued to be intermittently febrile and displayed worsening mental status. On the second day of hospitalization, the patient’s CSF and blood cultures were positive for *L. monocytogenes*, and her antibiotic regimen was changed to ampicillin and gentamicin. Her fevers resolved, her mental status and symptoms improved, and she was discharged from the hospital on day eight after a peripherally inserted central catheter was placed to receive a total of 21 days of ampicillin. The patient did not have recurrence of her Crohn’s symptoms while hospitalized, and she was switched to methotrexate for outpatient therapy.

CPC-EM Capsule

What do we already know about this clinical entity?

Listeria monocytogenes *infections have been documented in patients taking infliximab, and require a tailored antibiotic regime for successful treatment*.

What makes this presentation of disease reportable?

*A pediatric patient on infliximab therapy developed Listeria meningitis despite a negative polymerase chain reaction assay of cerebral spinal fluid*.

What is the major learning point?

*Infliximab increases chances of opportunistic infections, and ampicillin should be added to initial empiric antibiotic coverage*.

How might this improve emergency medicine practice?

*Reduce delay to definitive antibiotic treatment in an immunocompromised patient*.

## DISCUSSION

There are few case reports in the literature of *L. monocytogenes* infection in an immunocompetent host; however, it is an agent to be suspected in the immunocompromised population. Many of the reported cases occur in adult patients receiving infliximab for the treatment of rheumatoid arthritis.^11^ Listeriosis is rarely documented in immunocompetent children,^2^,^9^,^14^ with iron overload being suggested as a potential risk factor.^9^

Three-month mortality in patients with bacteremia has been found to be 45%.^3^ Mortality increased when there was a delay in administration of appropriate beta-lactam inhibiting agents, such as ampicillin. Relapses have occurred in immunocompromised patients after 14 days of treatment,^7^ leading to recommendations of three to six weeks for the immunocompromised with bacteremia and four to eight weeks for those with central nervous system infections.

Gentamicin has been reported to have synergistic effects with ampicillin in the treatment of listeriosis. Trimethoprim-sulfamethoxazole has been used to treat listeriosis successfully^12^ in the case of penicillin allergy.

The PCR assay for the *hyl* gene, encoding listeriolysin O, has been reported to be specific and more sensitive than culture after antibiotic therapy has been initiated, with up to 10% of culture negative CSF having a positive PCR result in cases otherwise attributed to listeriosis based on clinical presentation and imaging results.^6^ The patient presented here is unique in that the CSF obtained at time of presentation resulted in a negative PCR assay but positive culture, demonstrating a rare false-negative PCR result.

It has been proposed that TNF-α plays an important role in the defense against infections.^15^ Infliximab, an anti-TNF-α agent, has found success in the treatment of inflammatory conditions such as rheumatoid arthritis and more recently Crohn’s disease. Clinicians should be aware of the increased risk of listeriosis and empirically cover as indicated for this not infrequently fatal infection regardless of initial PCR results.

## CONCLUSION

Patients receiving infliximab who display signs of central nervous system infection should be suspected to have *L. monocytogenes* as an infecting agent and empiric addition of ampicillin to their antibiotic regimen should be considered, with substitution of trimethoprim-sulfamethoxazole in cases of penicillin allergy, regardless of initial PCR results.
